# Coriloxin Exerts Antitumor Effects in Human Lung Adenocarcinoma Cells

**DOI:** 10.3390/ijms23073991

**Published:** 2022-04-03

**Authors:** Yu-Hsuan Kuo, Yi-Xuan Wang, Wan-Hua Peng, Nian-Yu Chi, Tzong-Huei Lee, Chi-Chung Wang

**Affiliations:** 1 Department of Oncology, Chi-Mei Hospital, Tainan 710402, Taiwan; beethovan@gmail.com; 2 Graduate Institute of Biomedical and Pharmaceutical Science, Fu Jen Catholic University, New Taipei City 242062, Taiwan; yulon180506@gmail.com (Y.-X.W.); peng0987peng0987@yahoo.com.tw (W.-H.P.); ft870304@gmail.com (N.-Y.C.); 3 Institute of Fisheries Science, National Taiwan University, Taipei 106319, Taiwan; thlee1@ntu.edu.tw

**Keywords:** lung adenocarcinoma, natural product, coriloxin, anticancer

## Abstract

Both in Taiwan and around the world, lung cancer is a primary cause of cancer-related deaths. In Taiwan, the most prevalent form of lung cancer is lung adenocarcinoma, a type of non-small-cell lung carcinoma. Although numerous lung cancer therapies are available, their clinical outcomes are unsatisfactory. Natural products, including fungal metabolites, are excellent sources of pharmaceutical compounds used in cancer treatment. We employed in vitro cell invasion, cell proliferation, cell migration, cell viability, and colony formation assays with the aim of evaluating the effects of coriloxin, isolated from fermented broths of *Nectria balsamea* YMJ94052402, on human lung adenocarcinoma CL1-5 and/or A549 cells. The potential targets regulated by coriloxin were examined through Western blot analysis. The cytotoxic effect of coriloxin was more efficiently exerted on lung adenocarcinoma cells than on bronchial epithelial cells. Moreover, low-concentration coriloxin significantly suppressed adenocarcinoma cells’ proliferative, migratory, and clonogenic abilities. These inhibitory effects were achieved through ERK/AKT inactivation, epithelial–mesenchymal transition regulation, and HLJ1 expression. Our findings suggest that coriloxin can be used as a multitarget anticancer agent. Further investigations of the application of coriloxin as an adjuvant therapy in lung cancer treatment are warranted.

## 1. Introduction

Over the past several decades, natural products have been established as excellent sources of pharmaceutical compounds used in cancer treatment. Approximately 75% of small molecules with clinical applications are either natural products or are derived therefrom [1]. In recent years, studies on naturally occurring lead compounds with components originating from terrestrial microorganisms, plants, extreme environments, marine environments, and fungal metabolites have been conducted [1,2]. However, fungi-derived compounds have yet to be approved as anticancer agents. Many fungi cannot be cultured, which make it more challenging to access their metabolite-producing potential [3]. Laboratory characterization and culture have been performed on less than 5% of fungal species. The potential antitumor activities of fungal species require urgent examination [4].

Over 6,600 highly diversified fungal species have been documented in Taiwan [5]. In 1886, *Trichobotrys effusa* (Berkeley and Broome) Petch was reported for the first time and classified as belonging to the phylum Deuteromycota [6]. A study observed that ethyl acetate extract from the fermented broth of *T. effusa* YMJ1179 inhibited the growth of A549 lung cancer cells [7]. Coriloxin, an antimicrobial metabolite, has been isolated from this fermented broth [8,9]. Furthermore, coriloxin has been isolated from various xylariaceous, endophytic fungi, including strains YUA-026, *Xylaria* sp. 101, and PB-30 [10,11,12]. An investigation revealed that endophytic fungi could provide various secondary metabolites that have diversified biological activities and structures [13]. Regarding the cytotoxicity of coriloxin, one study observed variation in the half-maximal inhibitory concentration (IC_50_) of coriloxin, depending on the type of human cancer cells [12]. However, whether coriloxin has other biological effects on cancer cells remains unclear.

In Taiwan, lung cancer is the second-most prevalent cancer form. Globally, this disease is the most frequent cause of cancer-associated mortality [14]. Patients with lung cancer often develop resistance to radiotherapy, chemotherapy, or targeted therapy, the mainstay treatments for lung cancer (along with surgery) [15]. Furthermore, these treatments do not yield favorable clinical outcomes. Therefore, developing new therapeutic approaches or agents that selectively kill lung cancer cells and inhibit metastasis without harming noncancer cells is essential for improving clinical outcomes and reducing resistance.

Mounting experimental evidence indicates that several natural products exert antitumor effects on lung cancer cells [16]. However, whether coriloxin has anticancer effects on lung cancer cells in general remains unclear. Herein, fermented broths of *Nectria balsamea* YMJ94052402 were used to obtain ethyl acetate extracts, from which coriloxin was then isolated. Subsequently, we evaluated the anticancer effects of coriloxin on human lung adenocarcinoma cells. Furthermore, the potential molecular mechanisms underlying these effects were explored.

## 2. Results

### 2.1. Suppression of Cell Viability and Cell Proliferation by Coriloxin

**Figure 1:**
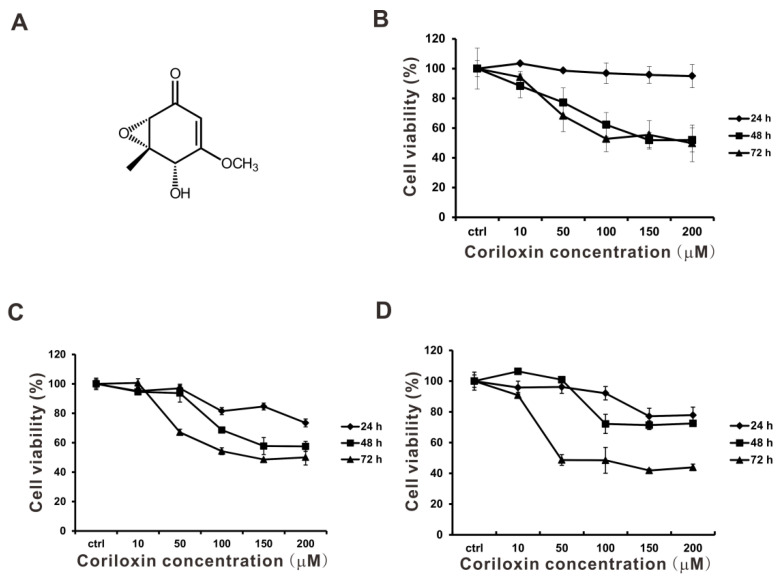
Influence of coriloxin on BEAS2B, A549, and CL1-5 cell viability. (**A**) Chemical composition of the isolated coriloxin. Following treatment with various concentrations of coriloxin, (**B**) BEAS2B, (**C**) A549, and (**D**) CL1-5 cells were subjected to the MTT assay. Results shown are percentages of the solvent control group (0.01% DMSO). These results are representative of two independent experiments performed, at least, in triplicate. Data are expressed as mean ±SD.

**Figure 2:**
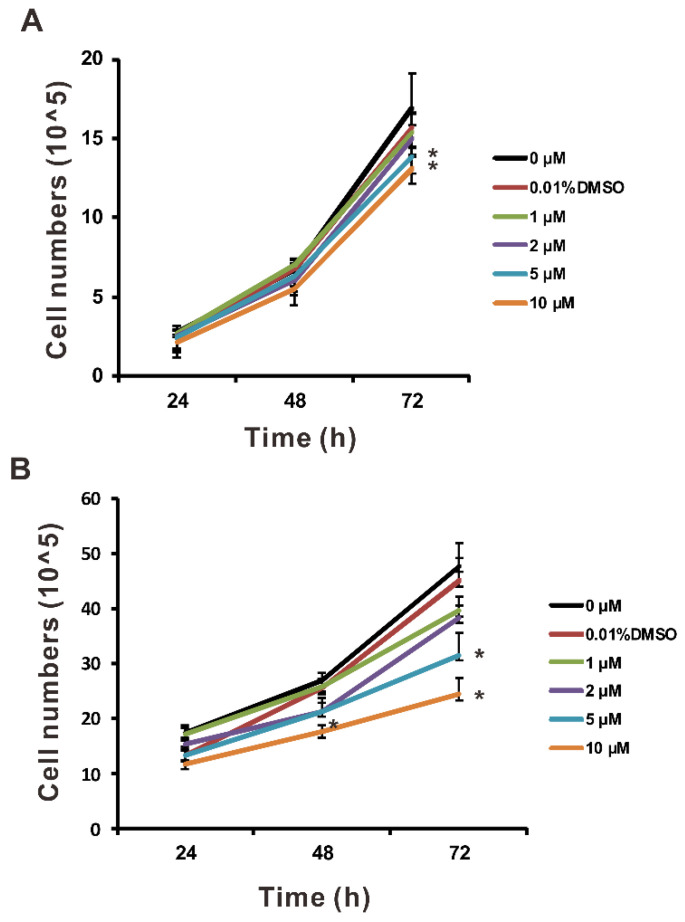
Antiproliferative effects of coriloxin on lung cancer cells. The proliferation activity of (**A**) A549 and (**B**) CL1-5 cells (*n* = 6 per group) treated with coriloxin for 24, 48, or 72 h was examined. Each experiment was performed, at least, in triplicate, and the values were reported as mean ± SD. * p < 0.05 indicates a significant difference from the solvent control group (0.01% DMSO).

Coriloxin (Figure 1A), which is derived from the mycoendophytic *Xylaria* sp. NBRTSB-20, is an antimicrobial metabolite [9]. However, evidence of its antitumor effects on lung cancer cells is scant. To study the effects of coriloxin on cell viability, we exposed BEAS2B, A549, and CL1-5 cells to indicated concentrations of coriloxin for 24, 48, and 72 h. According to the results of the MTT assay (Figure 1), coriloxin dose-dependently reduced the cellular viability of all three types of cells under the 72 h condition. The IC_50_ of coriloxin in the BEAS2B cells was 197.63 µM (Figure 1B). The cytotoxic effect of coriloxin was more efficiently exerted on the A549 and CL1-5 cells, for which the IC_50_ was 137.04 and 48.72 µM, respectively (Figure 1C,D). The cell proliferation assay, involving the trypan blue exclusion assay, was conducted on these two cell lines. This involved exposure to sublethal concentrations of coriloxin. As shown in Figure 2, 10 µM coriloxin had significantly inhibited A549 and CL1-5 cell proliferation after 24 h treatment. Following 72 h treatment with 5 or 10 µM coriloxin, A549 and CL1-5 cells exhibited significantly lower proliferative activity than did the solvent control (0.01% DMSO) and untreated group (0 µM). Because coriloxin demonstrated stronger antitumor activity in CL1-5 cells than in A549 cells, the CL1-5 cells were further investigated.

### 2.2. Coriloxin Inhibits Colony Formation in CL1-5 Cells

**Figure 3:**
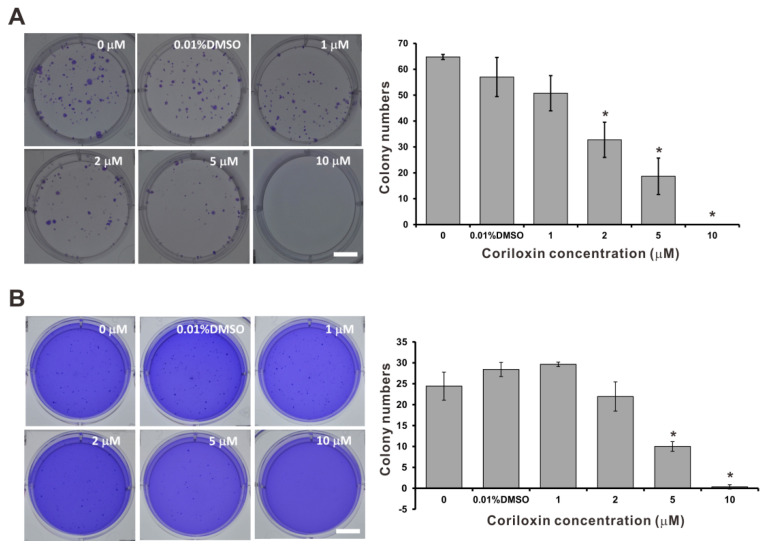
Anticlonogenic effects of coriloxin on CL1-5 cells. Representative images and integrated results of cells (*n* = 4 per group) under the (**A**) anchorage-dependent and (**B**) anchorage-independent clonogenic assays are presented. The graphs summarize the analytical results. Values are reported as means ±SD (*n* ≥ 3). * p < 0.05 indicates a significant difference from the solvent control group (0.01% DMSO). Scale bars, 1 cm.

The formation of anchorage-dependent CL1-5 colonies was dose-dependently inhibited by coriloxin (Figure 3A). Under treatment with 2 µM coriloxin, the colony count decreased significantly, to 82.4% that of the colony count in the solvent control group (*p* < 0.05). The formation of colonies was also significantly inhibited under treatment with 5 µM coriloxin. The anchorage-independent colony formation assay yielded the same results under the same concentration of coriloxin (Figure 3B). Under coriloxin concentrations exceeding 5 µM, CL1-5 cells were unable to form any colonies (Figure 3A,B).

### 2.3. Coriloxin Suppresses CL1-5 Cells’ Migration and Invasion Abilities

**Figure 4:**
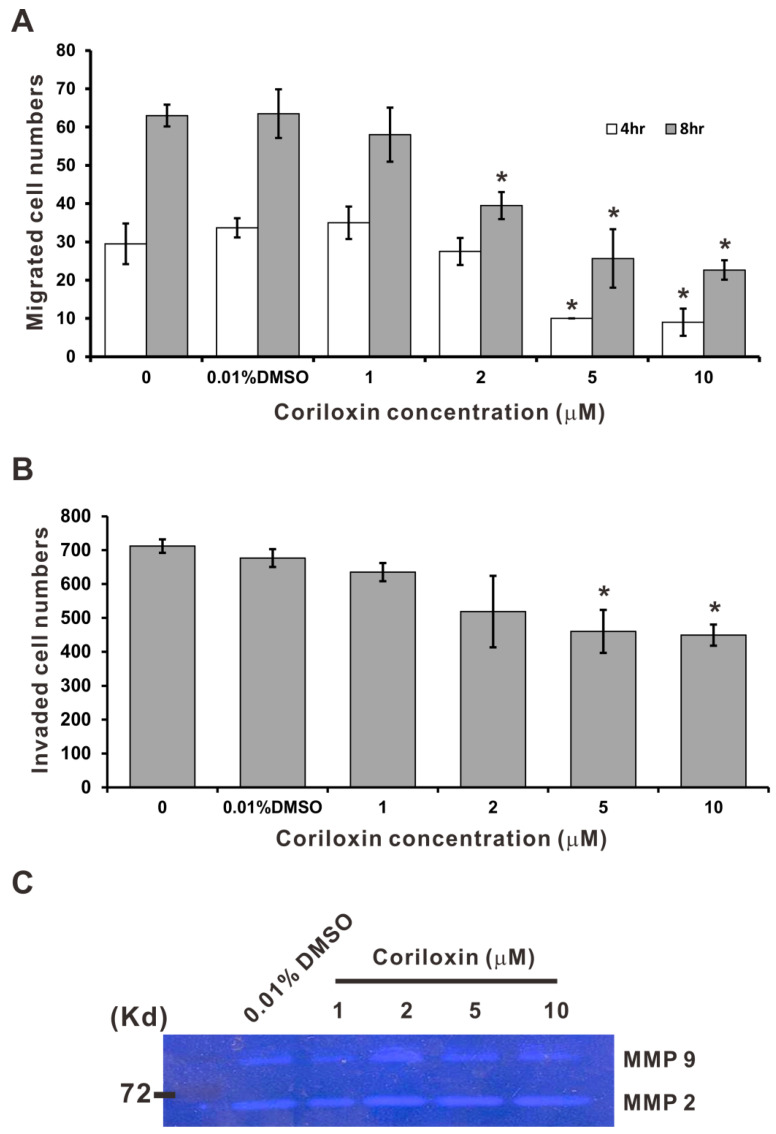
Antimigratory and anti-invasive effects of coriloxin on CL1-5 cells. (**A**) Wound-healing assay of the coriloxin-treated CL1-5 cells. We counted cells migrating into the wound area 4 and 8 h after wounding. (**B**) Cell invasion effects exerted by coriloxin (indicated concentrations). The data are representative of three independent experiments and are indicated as the mean ± SD. * p < 0.05 indicates a significant difference from the solvent control group (0.01% DMSO). (**C**) Gelatin zymography assay of MMP-2 and MMP-9 activities in coriloxin-treated cells.

We evaluated the effects of coriloxin on CL1-5 cell migration and invasion. Coriloxin was not discovered to have strongly affected the proliferative ability of CL1-5 cells after 24 h when the concentration was <10 µM (Figure 2B). Thus, a wound-healing assay was performed to evaluate the effects of coriloxin on CL1-5 cell migration at or under a concentration of 10 µM. As shown in Figure 4A, coriloxin dose-dependently inhibited the migration of CL1-5 cells. The Transwell membrane assay demonstrated that coriloxin dose-dependently reduced the cells’ invasive ability. Under treatment with 5 µM, the invasive ability of CL1-5 cells dropped significantly, to 67.9% of that for control cells (*p* < 0.05; Figure 4B). In sum, CL1-5 cells’ invasive and migratory abilities were suppressed under low-concentration coriloxin treatment. Dysregulation of matrix metalloproteinase (MMP) activity contributes to the metastasis of cancer cells [17]. As displayed in Figure 4C, the gelatin zymography assay revealed that coriloxin did not significantly influence MMP-2 or MMP-9 activity in CL1-5 cells. From the findings, we can infer that any suppressive effects of coriloxin on the invasive ability of lung cancer cells are exerted independently of MMP-2 and MMP-9 activities.

### 2.4. Putative Molecular Mechanisms of Coriloxin in Lung Adenocarcinoma Cells

**Figure 5:**
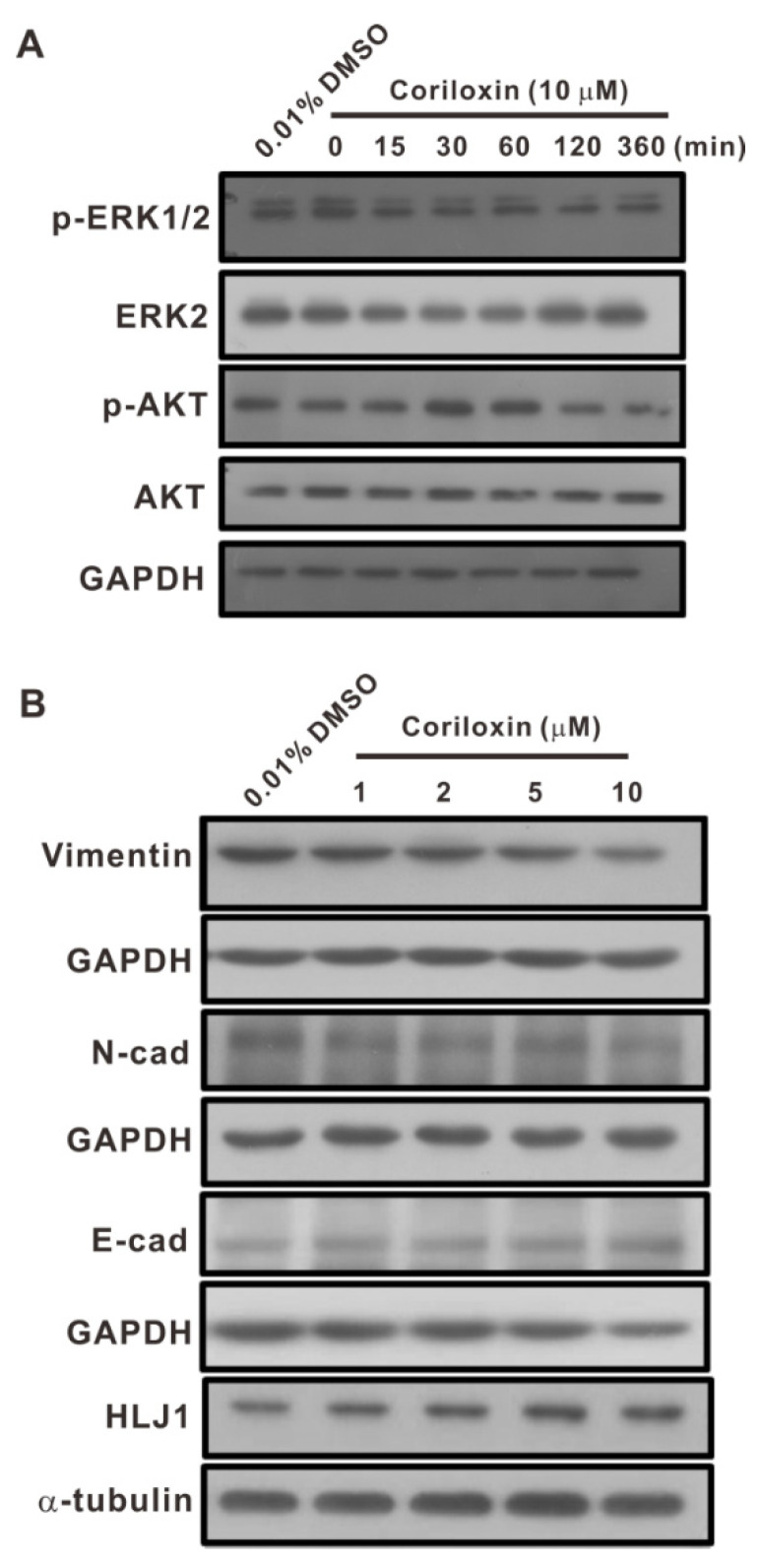
Effects of coriloxin treatment on ERK/AKT and the expression of EMT-related proteins in CL1-5 cells, as determined through Western blot analysis. (**A**) Effects of coriloxin on p-ERK1/2, ERK2, p-AKT, and AKT levels. Cells were treated with 10 µM coriloxin or 0.01% DMSO (solvent control), after which Western blotting revealed the protein expression levels. (**B**) Protein levels of EMT-related genes (Vimentin, N-cad, and E-cad) in CL1-5 cells subjected to 48 h coriloxin treatment (at indicated concentrations). The solvent control group was exposed to 0.01% DMSO. The loading controls were GADPH or α-tubulin. Each experiment was performed, at least, in duplicate.

We observed that CL1-5 cells’ proliferative, clonogenic, migratory, and invasive abilities were significantly suppressed by coriloxin. The signal transduction mechanisms underlying these effects have yet to be identified. A study suggested that the ERK1/2 pathway is involved in CL1-5 cell migration and proliferation [18]. On this basis, Western blotting was employed to assess coriloxin’s effect on the activation of AKT and ERK1/2 signaling. Following 15 min of treatment with 10 µM coriloxin, the cells’ p-ERK1/2 expression level was lower than in the control group (Figure 5A). Furthermore, 2 h coriloxin treatment also reduced the CL1-5 cells’ p-AKT levels. Aware that the epithelial–mesenchymal transition (EMT) contributes critically to cancer cell metastasis [19], we examined the levels of EMT-related markers in the cells. Coriloxin dose-dependently lowered the levels of vimentin and N-cad, which are mesenchymal markers (Figure 5B). Conversely, the post-treatment levels of epithelial marker E-cad were increased. *HLJ1*, a tumor suppressor gene implicated in non-small-cell lung cancer (NSCLC), was dose-dependently upregulated by coriloxin. The results suggest that, in lung cancer cells, several molecular mechanisms are involved in coriloxin’s antiproliferative, antimigratory, and anti-invasive effects.

## 3. Discussion

The high mortality rate among patients with lung cancer, the most globally prevalent malignancy, is attributable to cell metastasis. Various compounds derived from natural products, such as fungal metabolites, have been observed to reduce the aggressiveness of cancer cells through antitumor activity, for example, through decreasing cancer cell migration activities [16,20,21]. Although coriloxin has been isolated from several fungal species, whether coriloxin has any antitumor effects on human lung cancer cells remained unclear until now. This study is the first to report that coriloxin exerts antiproliferative, anticlonogenic, antimigratory, and anti-invasive effects on lung cancer cells. The anticancer activity of coriloxin was demonstrated through its reduction of p-ERK, p-AKT, and mesenchymal marker levels, as well as through its upregulation of E-cad and HLJ1 expression. The findings suggest that coriloxin has potential for application to cancer treatment as a multitarget agent.

Recent study has observed antimicrobial, anti-inflammatory, antiviral, and anticancer activities in natural products from endophytic fungi [3,4]. For example, leptosphaerone C, a polyketide isolated from the endophytic fungus *Penicillium* sp. JP-1, was cytotoxic to A549 cells (IC_50_: 1.45 µM) [22]. Herein, the cytotoxic effect of coriloxin was more efficiently exerted on lung adenocarcinoma cells than on bronchial epithelial cells. Exposure to sublethal concentrations of coriloxin resulted in the significant suppression of proliferative, invasive, and migratory activities. Although endophytes can produce secondary metabolites in vitro, the type and amount of the generated compound is affected by various factors, including the temperature of the culture, the degree of aeration, and the composition of the medium [23]. Herein, we successfully isolated coriloxin from ethyl acetate extracts derived from fermented broths of *N. balsamea* YMJ94052402.

Previous research has revealed that ERK and AKT pathways contribute crucially to cytoskeleton reorganization and to cell survival, proliferation, and differentiation [24,25]. According to one study, dysregulation of these pathways has a role in the progression of lung cancer, including its development and metastasis [24]. Therefore, targeting components of these pathways is a promising strategy for the formulation of novel lung cancer therapies. Herein, coriloxin lowered the expression of both p-ERK1/2 and p-AKT. As mentioned, in CL1-5 cells, 15 min coriloxin treatment downregulated the expression of p-ERK1/2. Moreover, AKT phosphorylation was suppressed following 2 h of coriloxin treatment. We previously suggested that the motility and proliferation of NSCLC cells are regulated by the ERK-signaling pathway [18]. Herein, coriloxin was effective in suppressing lung cancer cells’ proliferative, migratory, invasive, and clonogenic abilities. Prevention of the activation of ERK1/2 and AKT is a potential mechanism underlying these effects. Activated ERK1/2 refers to serine/threonine kinases that interact with various cytoplasmic and nuclear targets, leading to altered gene-expression patterns [26]. Further research regarding coriloxin’s antitumor effects on lung cancer cells is required to identify AKT- and ERK-targeted downstream target genes.

EMT contributes critically to the mobility, invasion, and metastasis of cancer cells [27]. N-cad and vimentin are well-known EMT markers, and their overexpression is frequently associated with the strengthening of cancer cells’ migratory and invasive abilities [28]. Herein, coriloxin treatment downregulated lung cancer cells’ vimentin and N-cad expression. Furthermore, coriloxin treatment increased the levels of E-cad and HLJ1. These findings are consistent with our previous findings [29,30]. HLJ1 overexpression suppressed the migratory, invasive, proliferative, and clonogenic abilities of lung cancer cells [29]. Another investigation indicated that HLJ1 regulates the invasion and migration of lung cancer cells by upregulating E-cad [30]. Thus, the present results suggest that HLJ1 regulation mediates coriloxin’s antitumor effects on lung cancer cells, at least partially.

As mentioned, this study is the first to demonstrate the antitumor effects of coriloxin on lung cancer cells. We observed that coriloxin suppresses the activation of ERK and AKT before downregulating HLJ1 expression. The results suggest that coriloxin has potential for application to cancer treatment as a multitarget agent. This study has several unanswered questions and limitations. First, whether the effect of coriloxin on HLJ1 expression is mediated by transcriptional regulation remains unclear. Second, the MMP-independent, anti-invasive mechanism of coriloxin requires further investigation. Third, whether other compounds isolated together with coriloxin from fungal fermented broths also exerted anticancer effects has yet to be determined. Fourth, whether coriloxin exerts antitumor effects on other types of cancer cell merits investigation. Finally, we only conducted in vitro experiments; the effects of coriloxin should be examined in vivo, for example through metastasis and tumorigenesis assays. Our findings serve as a reference for the development of new drugs—for example, coriloxin analogs—that can exhibit anticancer activities in lung cancer cells.

## 4. Materials and Methods

### 4.1. Fungal Fermentation

*N. balsamea* YMJ94052402 was isolated and identified by Dr. Yu-Ming Ju, Academic Sinica, Taipei, Taiwan [31]. The fungal strain was fermented at room temperature for 30 days in a 5 L serum bottle in which 20 g of Bacto-malt extract (Sparks, MD, USA) and 3 L of water were placed.

### 4.2. Compound Isolation and Purification

Ethyl acetate was used to partition the fermented broths to yield dried crude extracts (9.8 g). The crude extracts were redissolved in methanol and separated at a 2.2 mL/min flowrate by using a Sephadex open column (inside diameter 2.8 cm × 68 cm). A thin-layer chromatography (TLC) system was employed to check each collected 24 mL fraction. For development, ethyl acetate/acetic acid (20:1, *v*/*v*) was used. We identified compounds with similar skeletons via dipping in vanillin-H_2_SO_4_. The fractions were divided into portions (I, II, and III). A silica column (inside diameter 2.8 cm × 25 cm), with mixtures of *n*-hexane, ethyl acetate, and methanol as eluents in gradient mode, was used to further separate portion II (fr. 11–16) at a 5 mL/min flow rate. The compositions of the 75 mL fractions were examined using the TLC system, yielding seven subportions. The fourth subportion, eluted using 100% ethyl acetate, was dried and recrystallized using methanol to afford coriloxin (1.5 g).

### 4.3. Structural Elucidation

The structure of coriloxin (Figure 1) was visualized through various spectral analyses, including one-dimensional and two-dimensional NMR, IR, MS, and single-crystal XRD (Supplementary Figures S1–S8). The results were compared with the reported findings [10].

### 4.4. Cell Culture

The cell lines used were BEAS2B (ATCC CRL-9609), a primary immortalized bronchial epithelial cell line, and A549 (ATCC CCL-185) and CL1-5, which are human lung adenocarcinoma cell lines [32]. Cells were maintained in RPMI-1640 (Life Technologies, Inc., Carlsbad, CA, USA) containing 10% heat-inactivated fetal bovine serum (FBS; Life Technologies) supplemented with 1% penicillin–streptomycin (Life Technologies). Incubation was conducted at 37 °C in a humidified atmosphere with 5% carbon dioxide.

### 4.5. Cell Cytotoxicity Assay

To evaluate the cytotoxic effects (including the IC_50_) of various concentrations of coriloxin, we conducted the thiazolyl blue tetrazolium bromide (MTT) assay (Sigma-Aldrich, St. Louis, MO, USA), which is described elsewhere [33]. In brief, the tested cells were seeded at a density of 4,000 cells per well and incubated for 24 h in culture medium. Once the cells had been cultured with 0.01% dimethyl sulfoxide (DMSO) as a solvent control or with various concentrations of coriloxin for 24, 48, and 72 h, cell viability was examined.

### 4.6. Cell Proliferation Assay

Cells seeded at 1 × 10^5^ cells/well were incubated for 24 h. Next, we treated them with 0.01% DMSO (as a solvent control) or with various concentrations of coriloxin for 1–3 days. We assessed cell proliferation through the trypan blue exclusion test, manually counting the viable cells visible in a hemocytometer chamber placed under an inverted light microscope.

### 4.7. Colony Formation Assays

We performed an assay of anchorage-dependent colony formation. We resuspended 200 CL1-5 cells in RPMI-1640 containing 10% FBS before seeding them in six-well plates. Every 2 to 3 days, the culture medium (containing either 0.01% DMSO or coriloxin in various concentrations) was changed. After 7–10 days of incubation, the media were removed, and the cells were washed and fixed with 4% paraformaldehyde. Subsequently, the cell staining with crystal violet (0.05%) was conducted. We precoated six-well plates with 0.7% agarose in RPMI-1640 containing 10% FBS (bottom layer) and seeded 1500 CL1-5 cells in 0.35% agarose/RPMI-1640 containing 10% FBS (top layer) in an anchorage-independent assay. Treatment with 0.01% DMSO or various concentrations of coriloxin was performed on the cells, which were grown in soft agar. After 3 to 4 weeks of incubation, the cells underwent crystal violet staining as described previously [33]. Using an inverted light microscope, we identified colonies exceeding 0.5 mm in diameter.

### 4.8. Cell Migration and Invasion Assays

The migratory ability of DMSO- and coriloxin-treated cells was assessed using the protocol of the wound healing assay, as described elsewhere [34]. The cells that had migrated into the zone previously empty of cells at indicated times were counted through microscopic observation. Through the transwell membrane assay, performed using a transwell membrane (pore size 8 μm; Corning Costar, Cambridge, MA, USA) coated with Matrigel (BD Biosciences, Franklin Lakes, NJ, USA) [34], the cells’ invasion ability was examined. After 18 h of incubation, cells adhering to the polycarbonate filter’s lower surface were counted through light microscopy (magnification 200×) and subsequently photographed. We conducted the experiments in triplicate.

### 4.9. Gelatin Zymography Assay

At 5 × 10^5^ cells/well density, CL1-5 cells were seeded in six-well plates. Next, they were exposed to 0.01% DMSO (as a solvent control) or to various concentrations of coriloxin in serum-free media and cultured for 24 h. Sample preparation involved neither reduction nor boiling. Subsequently, sodium dodecyl sulfate–polyacrylamide gel containing 0.1% gelatin (Sigma-Aldrich) was used to conduct electrophoresis as described previously [33].

### 4.10. Western Blot Analysis

The levels of the affected proteins in the CL1-5 cells following coriloxin treatment were examined through Western blotting using the method described elsewhere [30]. The primary antibodies for phosphorylated ERK1/2 (p- ERK1/2), ERK2, phosphorylated AKT (p-AKT), AKT, E-cadherin (E-cad), N-cadherin (N-cad), vimentin, and HLJ1 were obtained from Santa Cruz Biotechnology (Dallas, TX, USA). The loading controls were anti-GADPH or anti-α-tubulin (both monoclonal antibodies). We incubated the membranes with the primary antibodies and subsequently washed them three times with a Tris-buffered saline–Tween 20 mixture. We then incubated them with secondary antibodies conjugated by horseradish peroxidase (Santa Cruz Biotechnology). Using an enhanced chemiluminescence detection system (GE Healthcare, NJ, USA), the membranes were finally examined.

### 4.11. Statistical Analysis

Triplicate experiments were conducted, and results are presented as means ± standard deviations. Significant differences (*p* < 0.05) were evaluated through analysis of variance conducted using Microsoft Excel software.

## 5. Conclusions

In summary, this study is the first to demonstrate that coriloxin isolated from fermented broths of fungal species can exert significant anticancer effects on human lung adenocarcinoma cells. The comprehensive molecular mechanisms governing the regulation of coriloxin in lung cancer cells warrant further investigation. Our results may open new perspectives concerning the development of coriloxin analogs or derivatives as potential primary components of chemotherapeutics for lung cancer treatment.

## Data Availability

Not applicable.

## Supplementary Materials

The following are available online at https://www.mdpi.com/article/10.3390/ijms23073991/s1.
