# Supplementary material for: Coriloxin Exerts Antitumor Effects in Human Lung Adenocarcinoma Cells
Source: Int J Mol Sci. 2022 Apr 3;23(7):3991. doi: 10.3390/ijms23073991 (PMC8999459; doi:10.3390/ijms23073991)
Supplement: Supplementary file 1 [file ijms-23-03991-s001.zip › ijms-1661278-supplementary.pdf]

## Supplementary Materials:

# Coriloxin Exerts Antitumor Effects in Human Lung Adenocarcinoma Cells

Yu-Hsuan Kuo, Yi-Xuan Wang, Wan-Hua Peng, Nian-Yu Chi, Tzong-Huei Lee, Chi-Chung Wang

**Correspondence:** Chi-Chung Wang, Ph.D., Graduate Institute of Biomedical and Pharmaceutical Science, Fu Jen Catholic University, No. 510, Zhongzheng Rd., Xinzhuang Dist., New Taipei City, 242062, Taiwan (R.O.C.) Phone: 886-2-29052039, Fax: 886-2-29053415, Email: 075006@mail.fju.edu.tw.

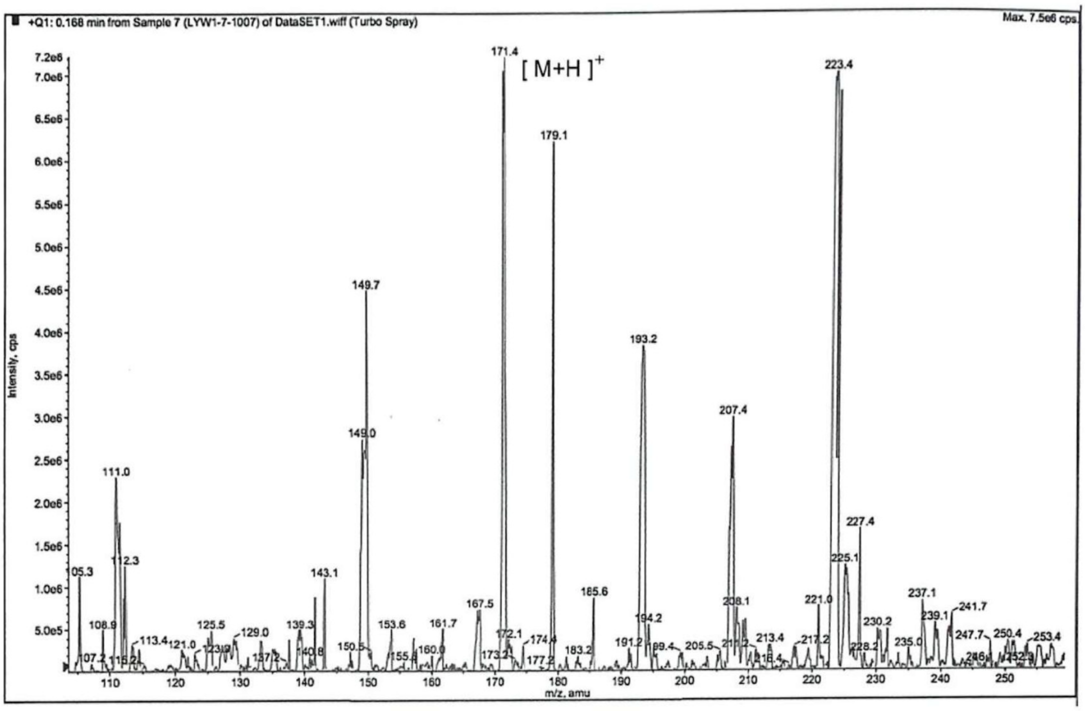


**Supplementary Figure S1. Low resolution ESI/MS of coriloxin.**

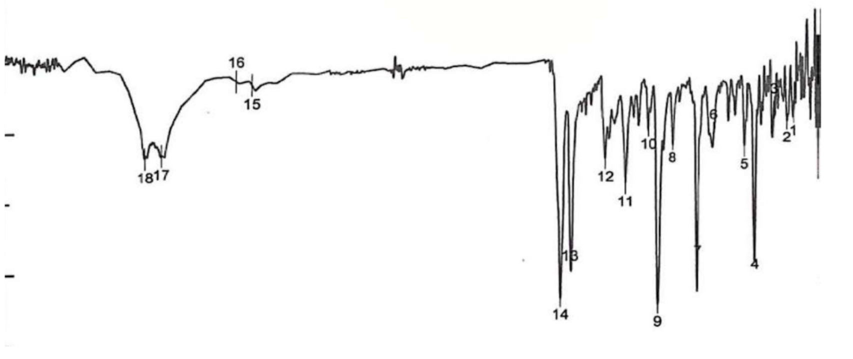


| NO. | Wavenumber [cm$^{-1}$] |
|---|---|
| 1 | 664.357 |
| 2 | 692.32 |
| 3 | 743.424 |
| 4 | 827.312 |
| 5 | 872.631 |
| 6 | 1001.84 |
| 7 | 1069.33 |
| 8 | 1176.36 |
| 9 | 1238.08 |
| 10 | 1277.61 |
| 11 | 1373.07 |
| 12 | 1457.92 |
| 13 | 1609.31 |
| 14 | 1648.84 |
| 15 | 2959.23 |
| 16 | 3026.73 |
| 17 | 3336.25 |
| 18 | 3403.74 |

**Supplementary Figure S2. IR spectrum of coriloxin.**

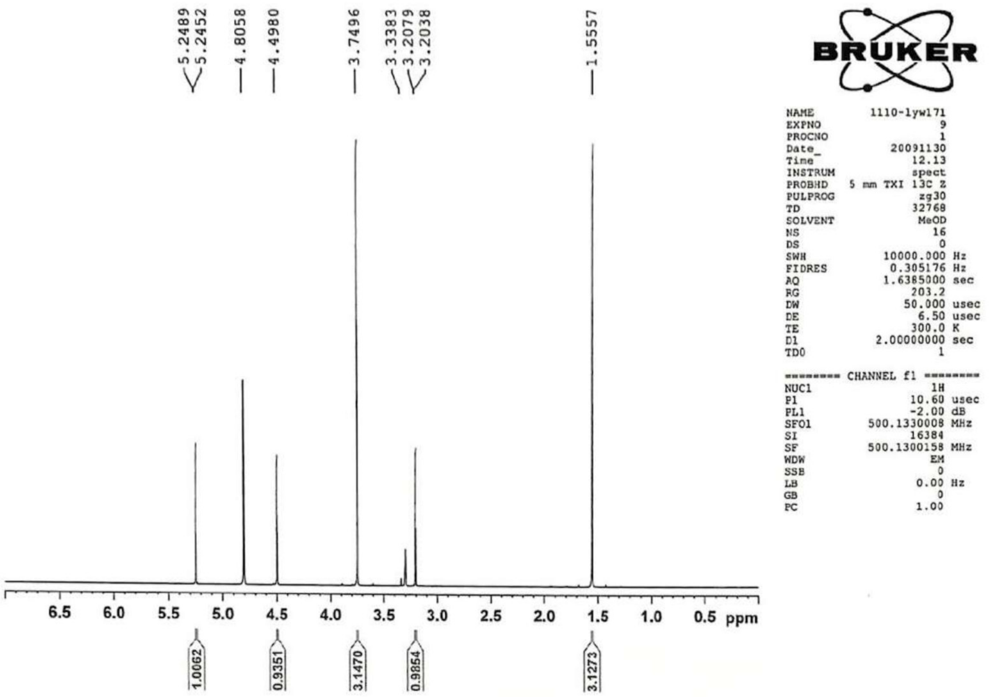


**Supplementary Figure S3. $^{1}$H NMR spectrum of coriloxin.**

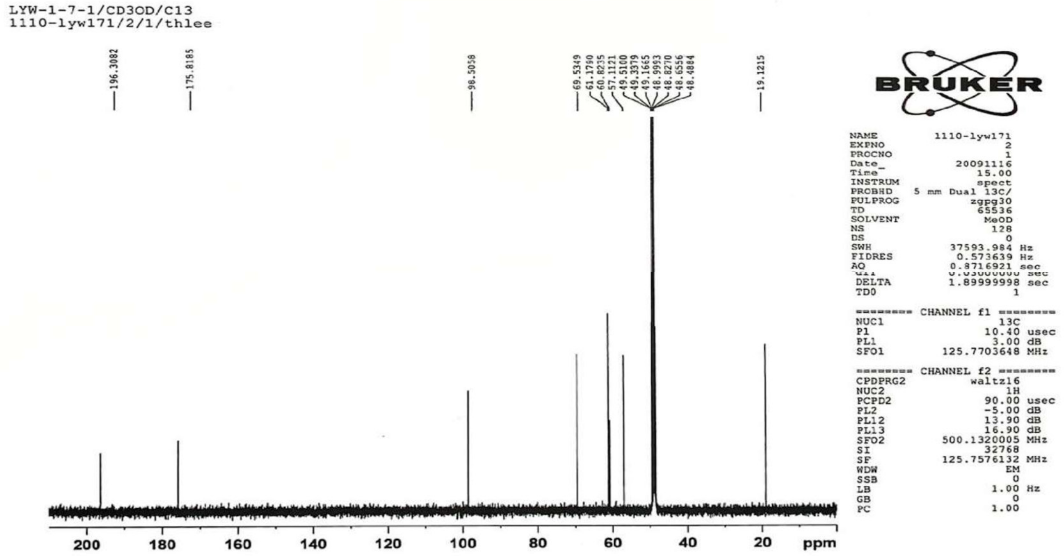


**Supplementary Figure S4. $^{13}$C NMR spectrum of coriloxin.**

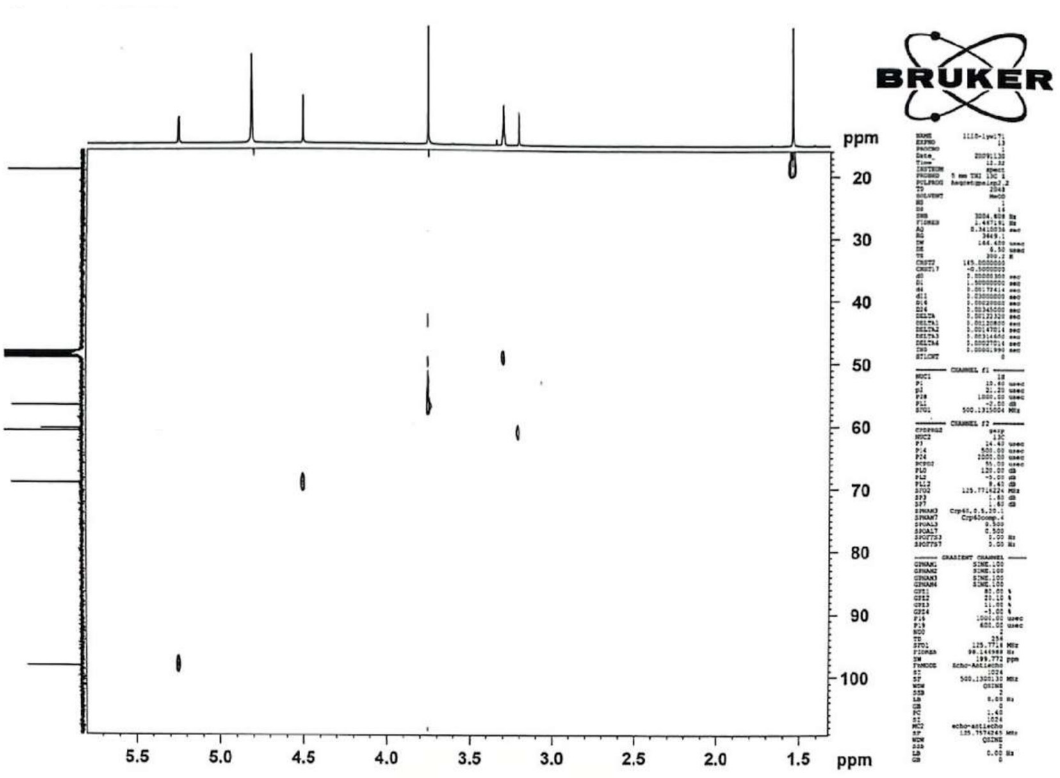


**Supplementary Figure S5. HSQC spectrum of coriloxin.**

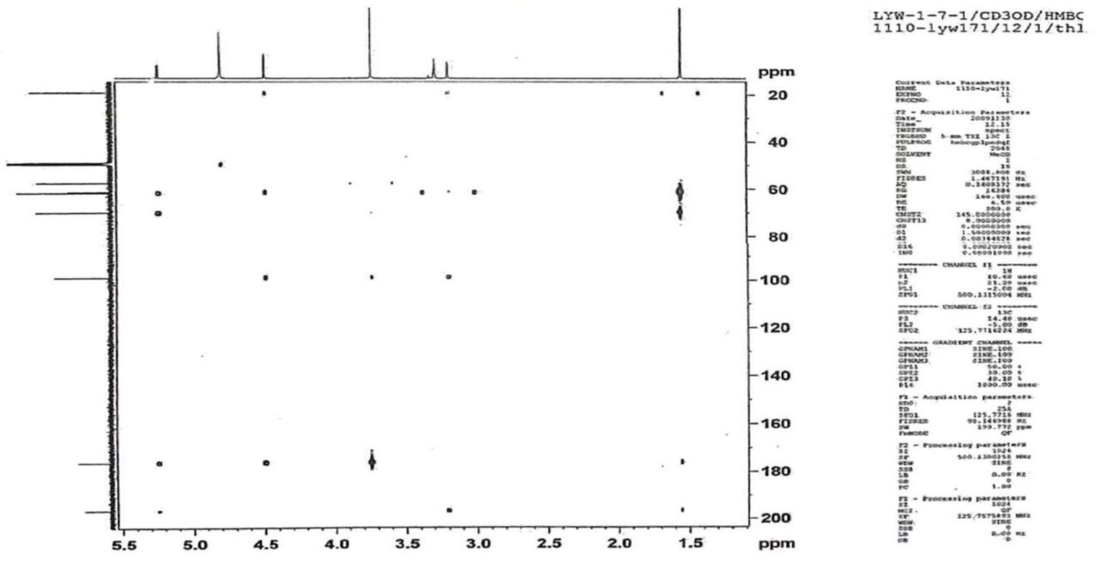


**Supplementary Figure S6. HMBC spectrum of coriloxin.**

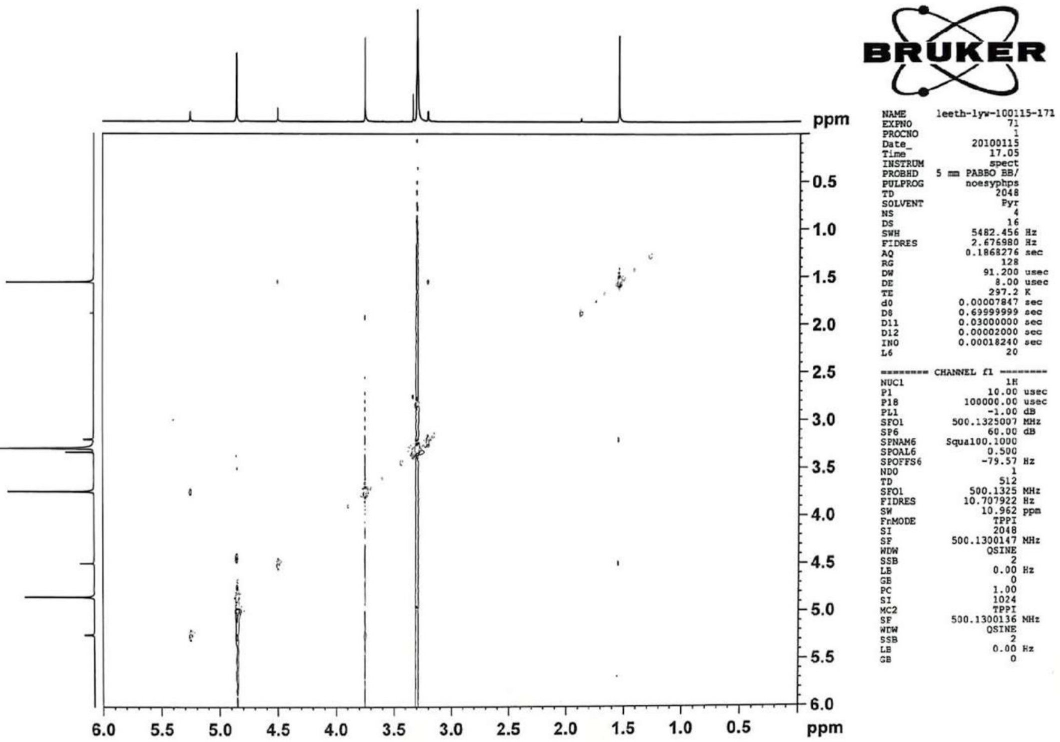


**Supplementary Figure S7. NOESY spectrum of coriloxin.**

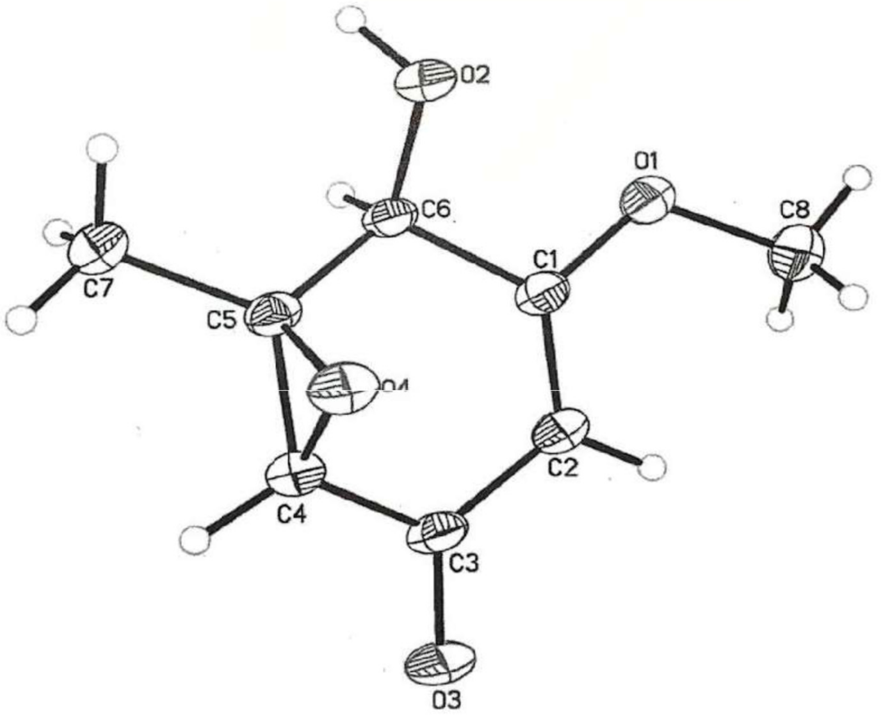


**Supplementary Figure S8. Computer-generated perspective drawings of the X-ray crystallographic model of coriloxin.**
